# Supplementary material for: Rapid assay development for low input targeted proteomics using a versatile linear ion trap
Source: Nat Commun. 2025 Apr 23;16:3794. doi: 10.1038/s41467-025-58757-8 (PMC12015518; doi:10.1038/s41467-025-58757-8)
Supplement: Supplementary file 1 — Supplementary Information [file 41467_2025_58757_MOESM1_ESM.pdf]

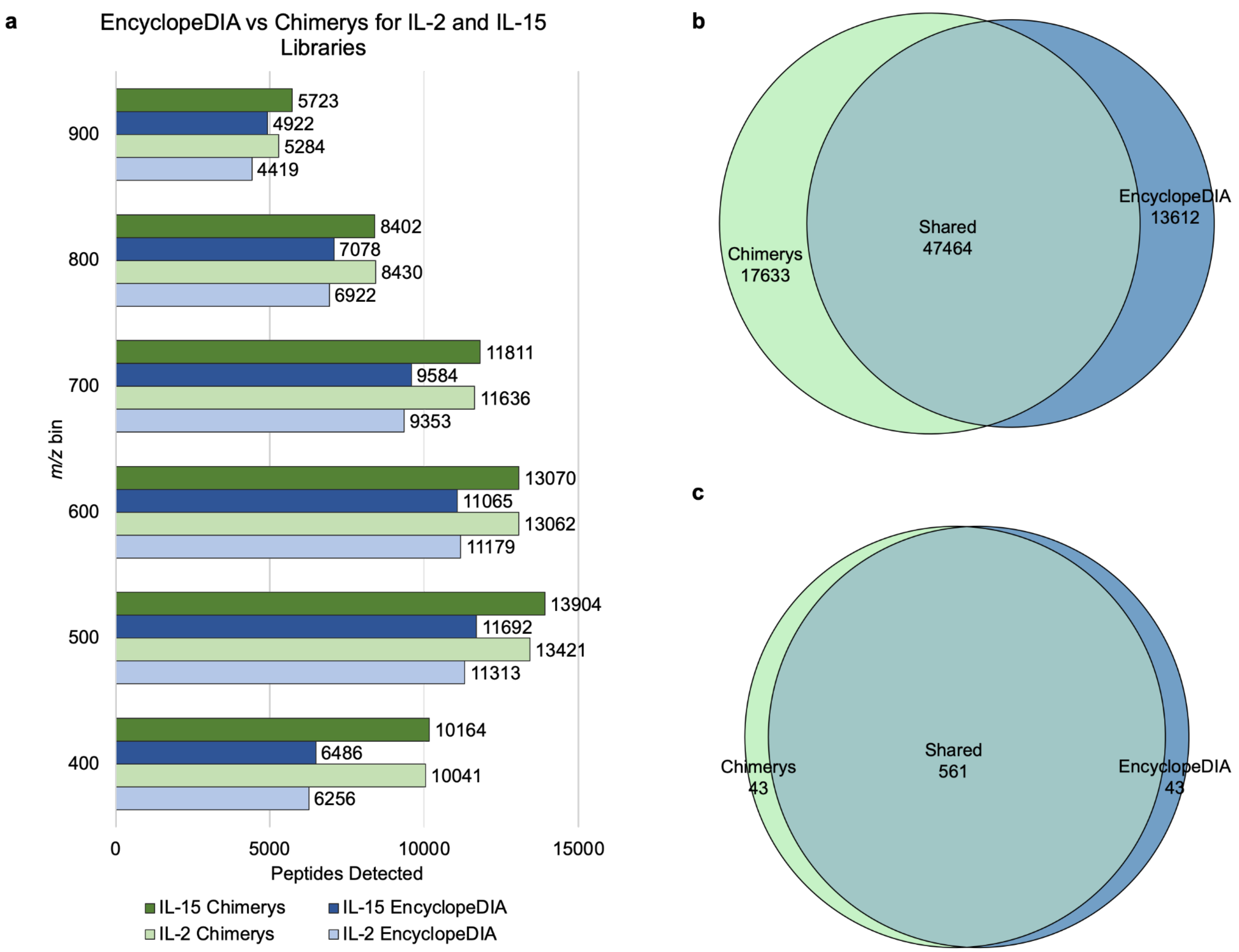

Supplementary Figure 1.
Comparison of Chimerys and EncyclopeDIA detections for chromatogram libraries. (**a**) displays peptide detections in hIL-2 and hIL-15 stimulated T cell libraries using 6× gas phase fractionated DIA injections. Both (**b**) and (**c**) comprise peptides detected in the IL-2, IL-15, and pooled libraries. (**b**) The comparison in software detections for a global library between Chimerys, EncyclopeDIA, and the shared number of peptides detected. (**c**) The comparison in software detections between Chimerys and EncyclopeDIA for validated targets of the 10, 20, and 50 peptide/cycle PRM assays.

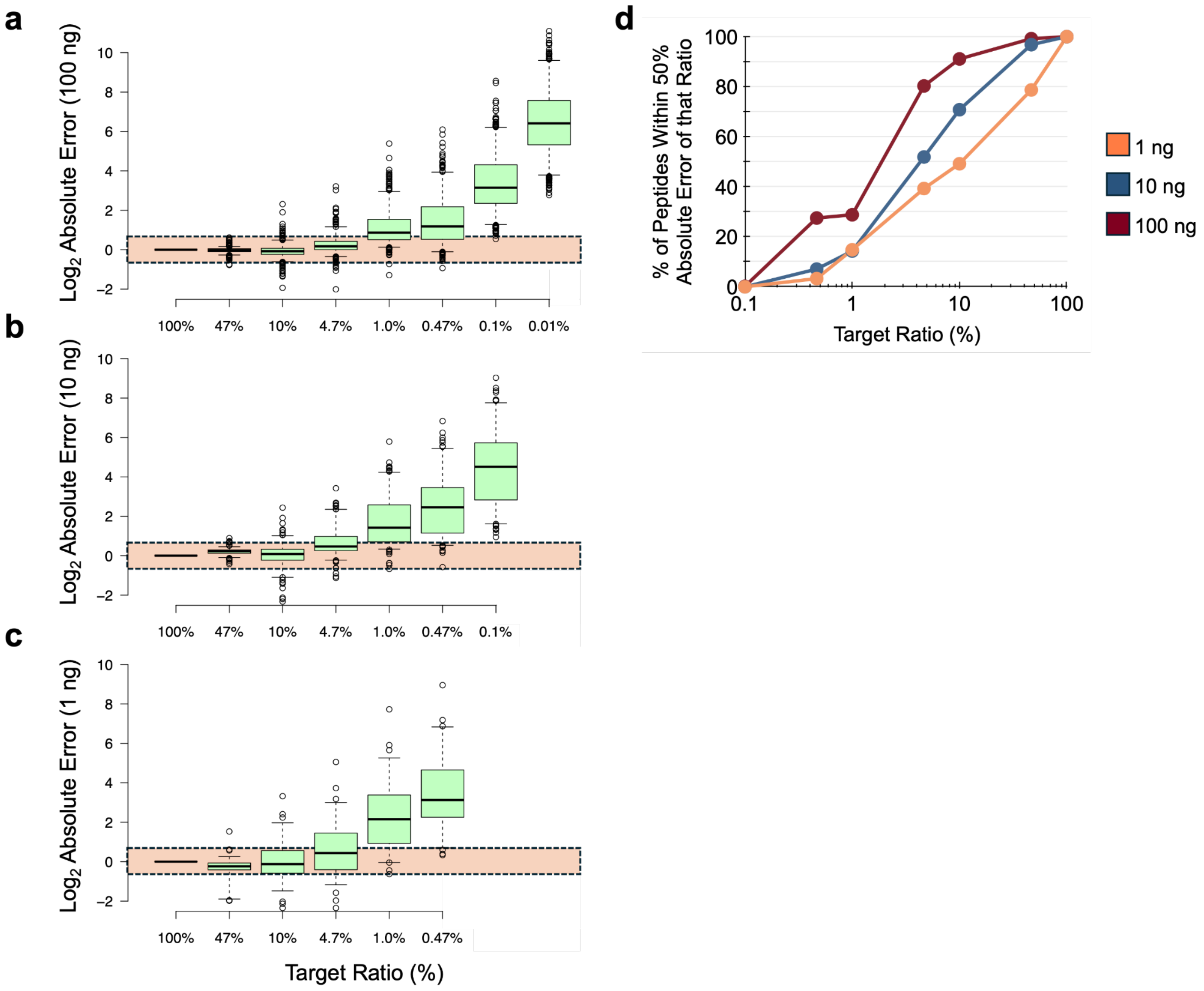

Supplementary Figure 2.
Absolute error levels for the Q-LIT matrix-match calibration curve at (a) 100 ng, (b) 10 ng, and (c) 1 ng of material on-column. The pink band represents ±50% absolute error on the estimated ratio. The acceptable error band shrinks as target ratios become smaller, making it harder to hit precisely. For example, the pink band at 10% indicates 5% to 15%, while the pink band at 1% indicates 0.5% to 1.5%. (d) The percent of peptides within the pink band at a target ratio.

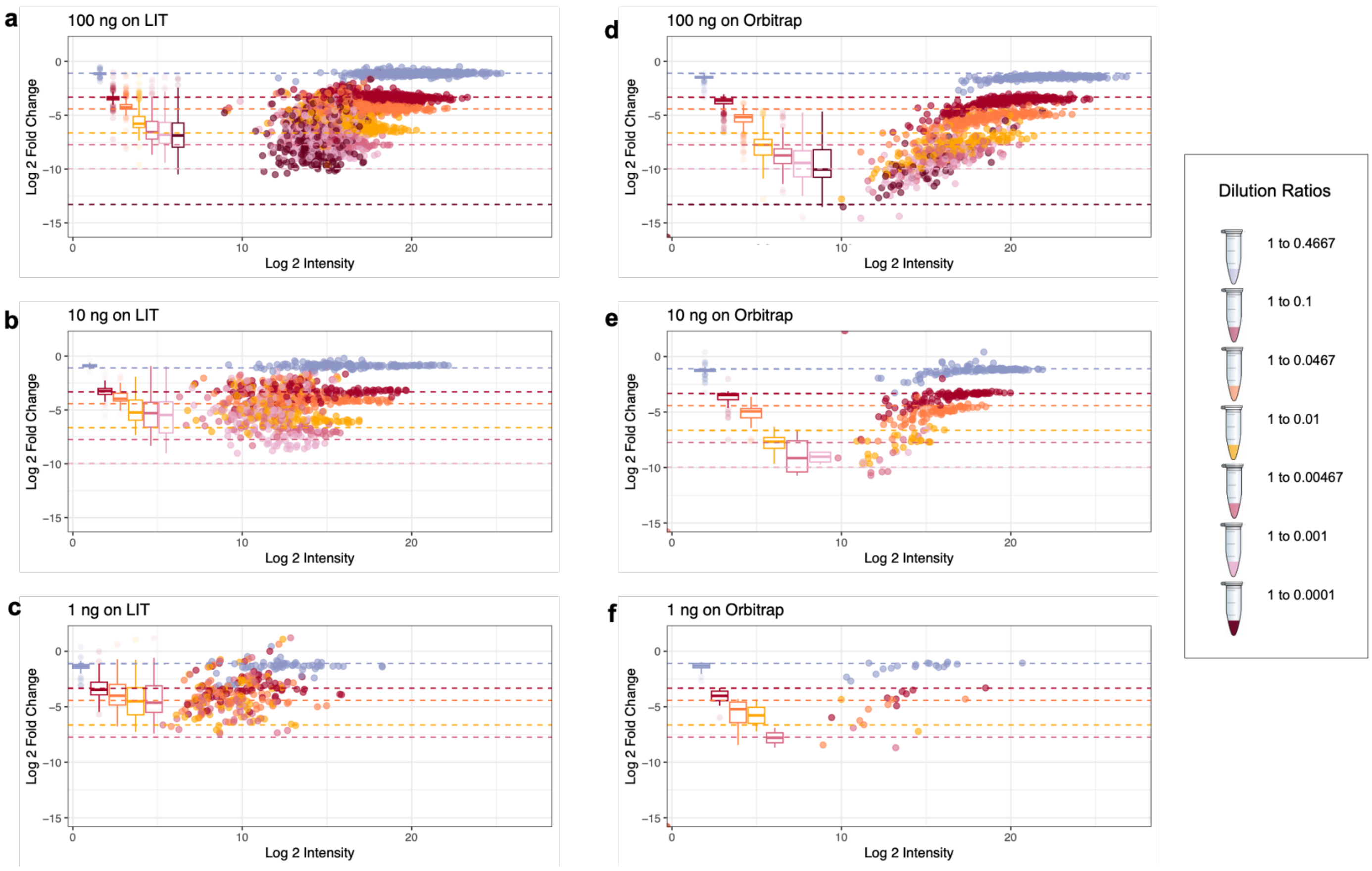

Supplementary Figure 3.
Quantitative accuracy of PRM intensities acquired using 50 peptides per cycle, 20 peptides per cycle, and 10 peptides per cycle assays on an LIT (**a, b** and **c**) and Orbitrap (**d, e,** and **f**)**.** Peptides were diluted in a pool of dimethyl-labeled background peptides over several orders of magnitude, specified in **Supplemental Table 1**. While both analyzers have comparable quantitative accuracy over 100, at 10, and 1 ng, the quantitative accuracy spans a larger dynamic range on the Orbitrap compared to the LIT. This is noted in (**a**) and (**d**) by the alignment of the acquired signal compared to the expected signal down over at least three orders of magnitude. This figure emphasizes the types of quantitative errors observed by both instruments. The Q-LIT tends to measure higher values where there are small signals (as indicated by the box plots shifting up at lower dilution ratios) due to higher interference caused by lower resolution. In contrast, the Q-Orbitrap tends to measure 0 when there are small signals (as indicated by the box plots shifting down at lower dilution ratios) due to the limited capacity of the Orbitrap.

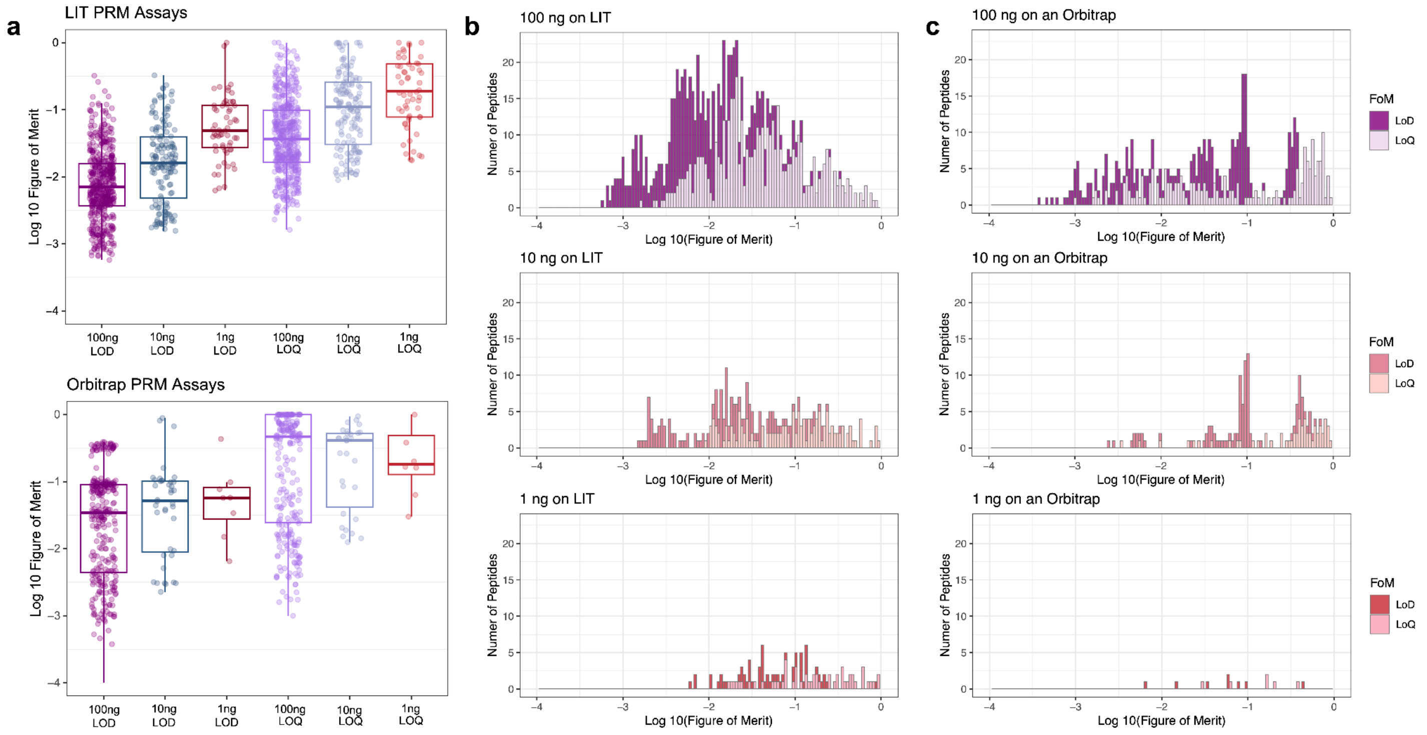

Supplementary Figure 4.

The distribution of figures of merit (FoM) on the Q-Orbitrap compared to the Q-LIT, calculated from PRM calibration curves. (**a**) The FoM for the same peptides on the Orbitrap and LIT. The Orbitrap assays were scheduled using the peptides observed on the LIT to allow for direct comparison in the same biological matrix. The Orbitrap assays did not observe all peptides detected with the LIT, partially due to a lack of sensitivity. As a result, there are a greater number of zero values for the FoM in the Orbitrap. (**b-c**) Histograms displaying the distribution between FoM on the LIT (**b**) and Orbitrap (**c**) at 1, 10, or 100 ng of peptides per infection. As the number of peptides decreases with a decreasing amount on column, the distance between the median LoD and LoQ increases for both analyzers. (**c**) The double hump distribution on the Q-Orbitrap is likely caused by having zero values at the lower range of the curve rather than having noise values, as we do with the Q-LIT. Once the signal approaches the lowest point an Orbitrap can detect, or its “zero point,” EncyclopeDIA’s algorithm is forced to fit the curve around the existing last data points.

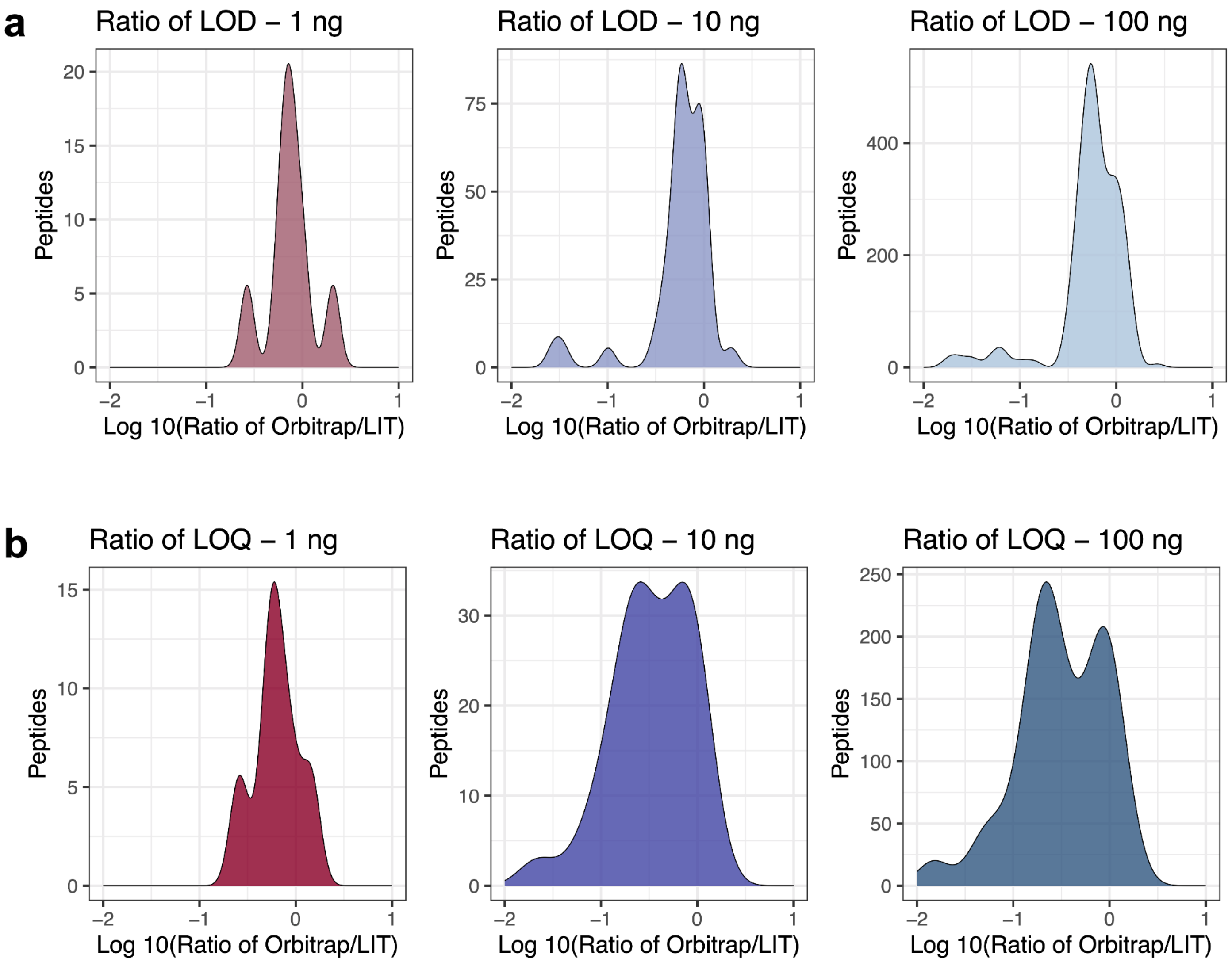

Supplementary Figure 5.

The peptide density of the ratio of the LoD (**a**) or LoQ (**b**) on a Q-Orbitrap compared to a Q-LIT. The overall density is centered between -1 and 0, likely due to the improved sensitivity of the LIT. Overall, the LoD and LoQ values are consistent on both the hybrid LIT and Orbitrap, matching previous data presented by Heil et al.[2] and Phlairaharn et al.[3] on the LIT within Tribrids.

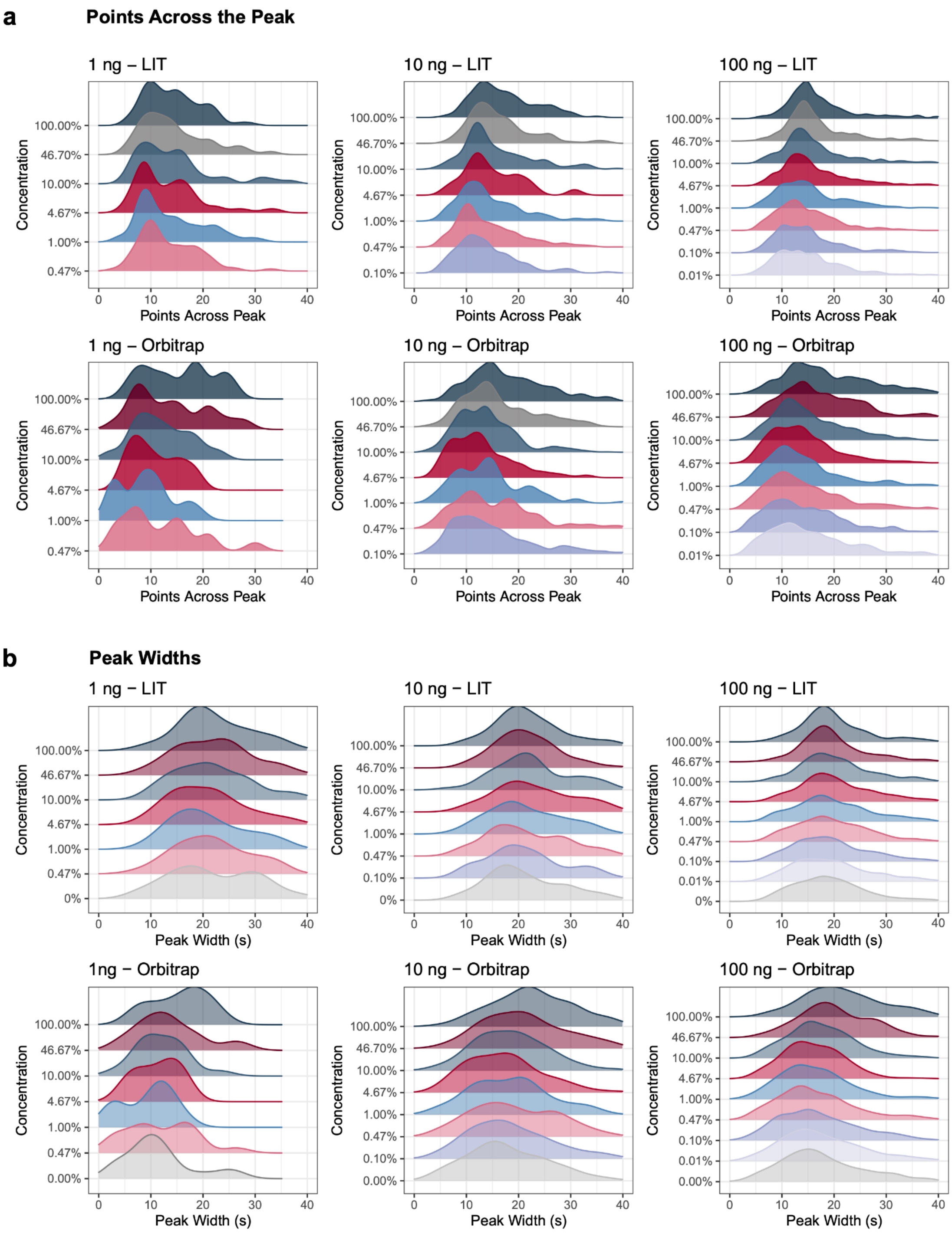

Supplementary Figure 6.

The (**a**) points across the peak and (**b**) peak widths for 1, 10, and 100 ng assays on the Q-LIT and the Q-Orbitrap. At 1 ng on the LIT, the median points across the peak were 11 points, while the Orbitrap had a much wider spred with a marginally higher median of 13 points across the peak. For both analyzers at 10 and 100 ng, all injections had a median of 14 points across the peak. (**b**) The average peak width in seconds for each assay. The peak width distributions are consistent across the Orbitrap (10 ng and 100 ng) and LIT (all assays), ranging from 18.6-21.6 seconds for the median peak width. At 1 ng on the Orbitrap, peak widths are a median of 12 seconds, likely due to the low signal observed at that amount with the Orbitrap analyzer.

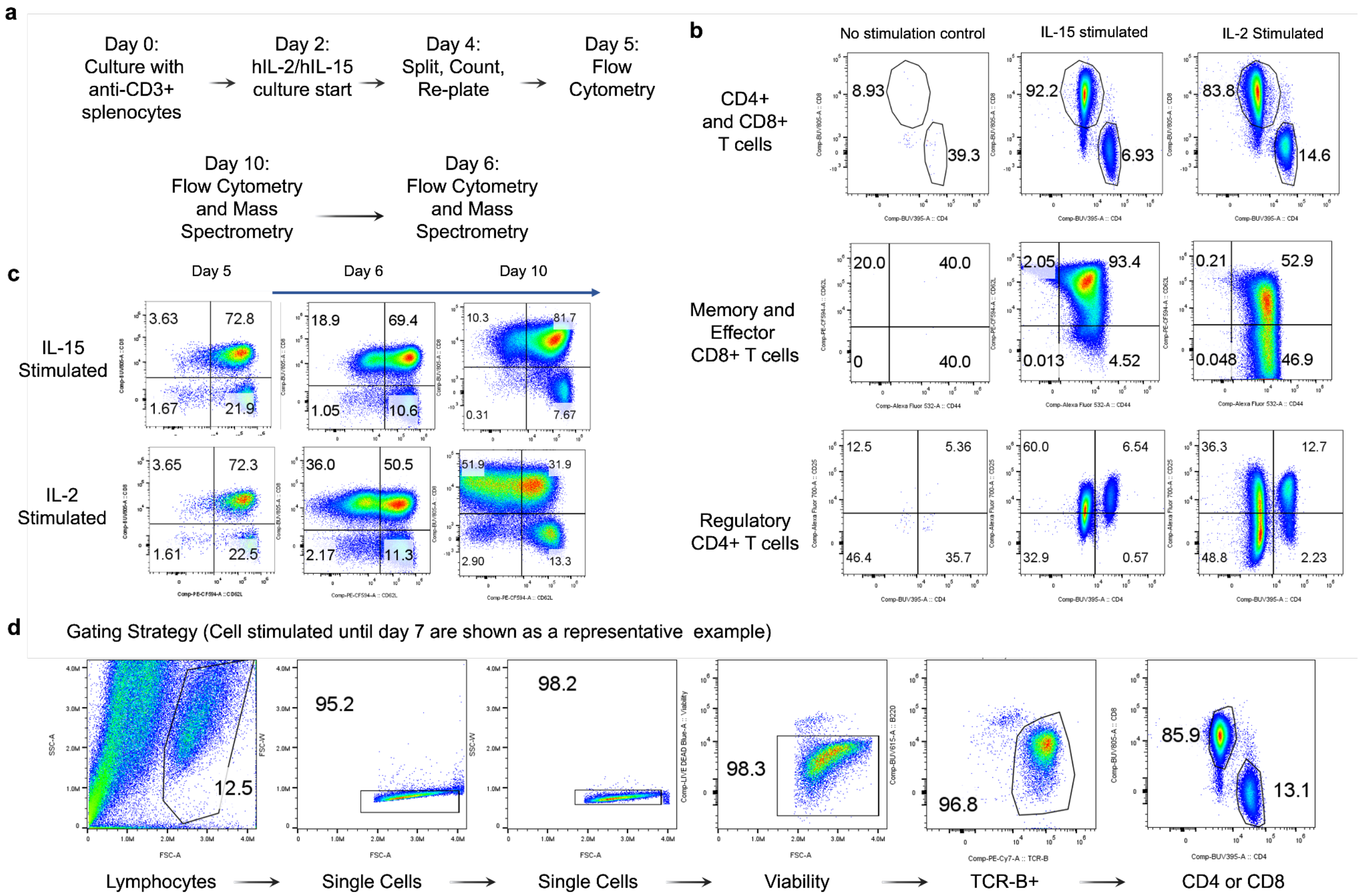

Supplementary Figure 7.

Additional flow cytometry validation data. (**a**) The workflow of the integrated flow cytometry and mass spectrometry analysis. (**b**) Flow cytometry plots displaying the populations of CD8+ and CD4^{+} T cells. Within the CD8^{+} T cell population of IL-2 and IL-15 stimulated cells, there are more CD44+ and CD62L+ cells in the IL-15 stimulated population than in the IL-2 stimulated population. (**d**) The gating strategy implemented for flow cytometry.

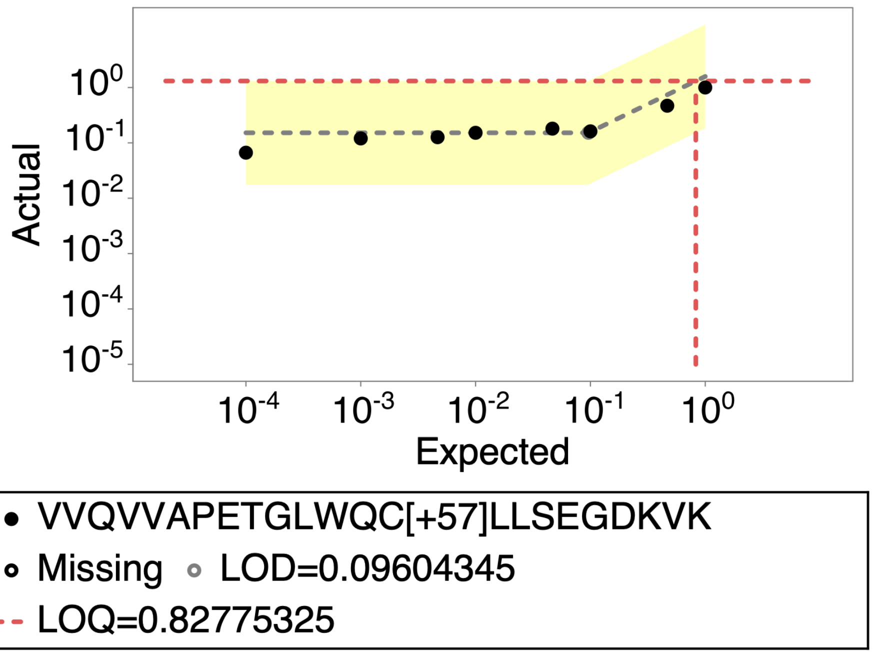

Supplementary Figure 8.

A peptide for CD4, which was assayed at the 1 and 10 ng levels. The following calibration curve is from the peptide VVQVVAPETGLWQCLLSEGDKVK and quantified in the Q-LIT PRM assay at the 10 ng level. The LoQ was estimated to be 0.8277525, which is above the signal for the 1:0 (ratio of foreground to background) injection, indicating that the LoQ was above 10 ng. The 1 ng assay on the Q-LIT did not quantify CD4, as this peptide was the only one observed for the protein in the translation library and fell outside the assay's dynamic range.

	instrument	m/z range	scan rate	mass resolution	dynamic range	sensitivity	release year
**LIT**	TFS Q-LIT (Stellar)	15–4 000	140 Hz<br>200 000 Da/s	0.5-2 FWHM	>1 x 10e5	atto- to femtomole	2024
**QqQ**	SCIEX QTRAP 6500+	50–2 000 (Trap),<br>5–2 000 (QqQ)	20 000 Da/s (Trap),<br>12 000 Da/s (QqQ)	0.7 FWHM	>1 x 10e5	atto- to femtomole	2016
	SCIEX Triple Quad 7500	5–2 000	20 000 Da/s	0.5 FWHM	>1 × 10e6	atto- to femtomole	2020
	Agilent 6495D	5–3 000	18 700 Da/s	0.4–2.5 FWHM	>6 × 10e6	atto- to femtomole	2023
	Shimadzu 8060NX	2–2 000	30 000 Da/s	0.5 FWHM	1 × 10e7	atto- to femtomole	2020
	TFS TSQ Altis	5–2 000	15 000 Da/s	0.2–2.0 FWHM	>1 × 10e6	atto- to femtomole	2019
**ToF**	Waters Synapt XS	20–64 000	30 Hz	75 000	1 × 10e4	femtomole	2019
	Bruker timsTOF SCP	50–20 000	120 Hz	60 000	5 × 10e4	zepto- to attomole	2021
	Bruker timsTOF HT	50–20 000	150 Hz	60 000	1 × 10e5	atto- to femtomole	2022
	Bruker timsTOF Ultra	50–20 000	300 Hz	60 000	5 × 10e4	zepto- to attomole	2023
	SCIEXZenoTOF 7600	40–40 000	133 Hz	42 000	1 × 10e5	atto- to femtomole	2021
**Orbi**	TFS Orbitrap Exploris	40–8 000*	40 Hz	480 000	1 × 10e5	femtomole	2019
	TFS Orbitrap Eclipse Tribrid	40–8 000	40 Hz (Orbitrap)<br>45 Hz (LIT)	1 000 000	>5 × 10e3	atto- to femtomole	2019
	TFS Orbitrap Ascend Tribrid	40–16 000*	45 Hz (Orbitrap)<br>50 Hz (LIT)	1 000 000	5 × 10e3	atto- to femtomole	2022
**AST**	Q-Orbitrap-Astral	40–2 000	200 Hz	80 000	>1 × 10e4	zepto- to attomole	2023

Supplementary Table 1.
A comparison of representative instruments broken down by analyzer type. Specifications are based on vendor reports. Table modified from Peters-Clarke et al.[1]

Marker	Channel	Fluorochrome	Vendor	Clone	Catalog	Concentration	Staining
**CD4**	UV2	BUV395	BD Biosciences	GK1.5	563790	7:40	Extracellular
**Viability**	UV6	Live/Dead Fix Blue	Thermo Fisher Scientific	-	L23105	17:40	Viability
**CD45R (B220)**	UV10	BUV615	Thermo Fisher Scientific	RA3-6B2	366-0452-82	7:40	Extracellular
**CD8**	UV16	BUV805	BD Biosciences	53-6.7	612898	7:40	Extracellular
**CD69**	V2	SuperBright 436	Thermo Fisher Scientific	H1.2F3	62-0691-82	4:20	Extracellular
**CD44**	B3	Alexa Fluor 532	Thermo Fisher Scientific	IM7	58-0441-82	11:00	Extracellular
**CD62L**	YG3	PE-CF594	BD Biosciences	MEL-14	562404	4:20	Extracellular
**TCR BETA**	YG9	PE-Cy7	Thermo Fisher Scientific	H57-597	25-5961-82	7:40	Extracellular
**CD25**	R4	Alexa Fluro 700	Thermo Fisher Scientific	PC61.5	56-0251-82	4:20	Extracellular

Supplementary Table 2.
The antibodies and staining conditions for the flow cytometry panel, including the fluorochrome, clone and concentration used for each. The antibodies for B220 and CD25 from ThermoFisher Scientific underwent advanced verification, according to the manufacturer's website. All other antibodies were verified using isoype controls by the manufacturers.

% Foreground	% Background	Amount of material for the 100 ng Calibration curve	Amount of material for 10 ng Calibration curve	Amount of material for 1 ng Calibration curve
100.00%	0.00%	100.000	10.000	1.000
46.67%	53.33%	46.667	4.667	0.467
10.00%	90.00%	10.000	1.000	0.100
4.67%	95.33%	4.667	0.467	0.047
1.00%	99.00%	1.000	0.100	0.010
0.47%	99.53%	0.466667	0.046667	0.004667
0.10%	99.90%	0.100	0.010	-
0.01%	99.99%	0.010	-	-

Supplementary Table 3.
Dilutions used for PRM calibration curves at 100 ng, 10 ng, and 1 ng of total material.

Supplementary Note 1.

Frequently Asked Questions (FAQ)

1. **How does the Stellar compare to other unit-resolution instruments?**
   As shown in supplemental Table 7, the Q-LIT instrument can scan more quickly than most previous Thermo Fisher Scientific (TFS) mass spectrometers, including the Q-Orbitrap LIT Ascend. Additionally, in Remes et al. paper titled "Hybrid Quadrupole Mass Filter – Radial Ejection Linear Ion Trap and Intelligent Data Acquisition Enable Highly Multiplex Targeted Proteomics,"[4] the authors compare figures of merit (limit of detection and limit of quantification) for the TFS TSQ Altis (QqQ analyzers) to the Stellar (Q-LIT) MS system. Specifically, in Figure 3 of Remes' work, they show the differences in detection for the Orbitrap Astral to the Stellar and the %CV of Astral vs Stellar PRM assays to show their similarity.

2. **What type of DIA isolation windowing scheme should be used with a Q-LIT? Why are staggered isolation windowing schemes not as beneficial on the Q-LIT platform?**
   Within this work, we used 8 *m/z* isolation windows across a mass range of 400-1000 *m/z* for wide-window injections. At 100 ng of HeLa with the maximum IIT set to 100 ms, the cycle time across 75 isolation windows was 2.8 seconds. This was the point at which we achieved a high enough number of detections with a reasonable duty cycle for sampling a minimum of 8-10 points across the peak. Using smaller isolation windows resulted in slightly more detections at the cost of lengthening the cycle time beyond a reasonable amount for accurate quantification.

   Additionally, with a "noisy" mass analyzer (such as a LIT, TOF, or Astral), we have found that staggered isolation windowing schemes typically do not improve the detection rate. In general, this is because the stagger demultiplexing algorithm used in Proteowizard (originally designed to process Orbitrap data) can produce "ghost" signals by over-interpreting noise.

3. **What are the differences between the LoD and LoQ obtained from a high-resolution instrument compared to a low-resolution instrument?**
   As the analyte signal drops with decreasing concentration, background interference can overwhelm the analyte signal. The point at which that background signal appears is referred to as the LoQ, where a change in signal directly reflects a change in analyte quantity. The point at which the background signal eclipses the analyte signal is the LoD, below which the analyte is not detectable. When calculating quantitative ratios, the lowest signal drops below the LoQ first, generally resulting in a regression towards 1:1. With regards to peptide calibration curves, the point at which the ratio becomes nonlinear indicates the LoQ.

   Instruments with low resolving power (LITs and quadrupoles) show more interference than instruments that can resolve small mass differences between ions, resulting in more background signals. In contrast, Orbitraps have limited trapping capacity, limiting the spectrum's dynamic range. In some cases, there are some peptides with low background signals that simply stop being measured before they fall below the LoQ. The LoQ can be challenging to estimate in this case since it is essentially below the LoD.

4. **How does the QR5 Plus quadrupole mass filter improve ion transmission on the Stellar?**
   The mass filter, located upstream of the LIT, is designed to increase ion transmission using a 5.25 mm field radius device. The field radius is the radial distance from the center of the quadrupole where the field strength is stable and uniform for ion transmission. When the field radius increases, the range of

stable ion trajectories is broadened, allowing for higher ion transmission. Decreasing the field radius size restricts the paths ions can take, consequently decreasing ion transmission. The 5.25 mm field radius is an improvement over the 4.0 mm field radius of the LIT in the Velos Pro and other modern Thermo ion traps.

5. **What version of EncyclopeDIA should be used to process ion trap data for sensitive quantification?**
   A tagged version of EncyclopeDIA titled “encyclopedia-4.7.11-executable.jar” is the best version to use for IonTrap/IonTrap data. This version can be found in the Ra Files on the panorama public page for this work, or on EncyclopeDIA’s bitbcket, under tagged versions in the source code. Here is a direct URL: https://bitbucket.org/searleb/encyclopedia/downloads/encyclopedia-4.7.11-executable.jar

Supplementary Note 2.

Tutorial for using PRM Scheduler embedded in EncyclopeDIA

This tutorial will outline how to use EncyclopeDIA to schedule parallel reaction monitoring (PRM) assays from a global GPF-DIA library and a desired list of proteins. The PRM Scheduler within EncyclopeDIA can accept preferred peptides, or a list of peptides which will preferentially be scheduled first. Additionally, an exclusion list of peptides can be specified as an input, which are peptides that will be excluded from the assay scheduled. The materials for this tutorial can be found on Panorama within the "Raw Data" tab, under the folder "PRMScheduler_Tutorial" at the following link: https://panoramaweb.org/StellarIonTrapForLowInput.url

Part A: Making a PRM assay with PRM Scheduler using a translation library

1. Select a list of proteins by functionally annotating proteins quantified using GPF-DIA. This can be done using GO terms, Reactome annotations, or specific pathways/biology of interest. This work used PANTHER DB to functionally annotate proteins of interest. We selected proteins related to immune pathways, biological processes, cytokines, transcription factors, and chaperones. The functional annotation table of the proteome acquired with DIA can be found in the **supplemental Excel sheet, tab S4**.

A	B	C	D	E	F	G	H	I	J	K	L
GeneID	MappedID	GeneName	PantherSubFan	PantherGO-Slir	PantherGO-Slir	PantherGO-Slir	PantherProtein	Pathway	Go-MF	Go-BP	Go-CC
MOUSE|MGI=I	Q09200	Beta-1,4 N-ace	BETA-1,4 N-ACI	UDP-glycosyltr	ceramide biosynthetic process(GO:0046513);glycosphingolipi(				(N-acetylneura	ganglioside bio	Golgi mem
MOUSE|MGI=I	Q3TFD2	Lysophosphatic	LYSOPHOSPHAT	O-acyltransferase activity(GO:(		endoplasmic re	acyltransferase(PC00042)		1-acylglycerol-3	lipid metabolic	endoplasm
MOUSE|MGI=I	Q9D517	1-acyl-sn-glyce	1-ACYL-SN-GLY(	O-acyltransferase activity(GO:(		endoplasmic re	acyltransferase(PC00042)		1-acylglycerol-3	lipid metabolic	nucleus(G(
MOUSE|MGI=I	Q61205	Platelet-activat	PLATELET-ACTI\	acetyltransferase activity(GO:0		cytoplasm(GO:	protein modifying enzyme(PC(		1-alkyl-2-acetyl	lipid metabolic	cytoplasm(
MOUSE|MGI=I	Q61206	Platelet-activat	PLATELET-ACTI\	acetyltransferase activity(GO:0		cytoplasm(GO:	protein modifying enzyme(PC(		1-alkyl-2-acetyl	lipid metabolic	fibrillar cer
MOUSE|MGI=I	Q80U44	Zinc finger FYV	ZINC FINGER FYVE DOMAIN-C(		endosomal tra	early endosome membrane(G(		TGF-beta signa	1-phosphatidyl	protein targeti	cytoplasm(
MOUSE|MGI=I	Q5SW28	Phosphoinositi	PHOSPHOINOS	kinase regulato	regulation of M	phosphatidylin	kinase modulat	Insulin/IGF pat	1-phosphatidyl	G protein-coup	nucleus(G(
MOUSE|MGI=I	Q8CHT0	Delta-1-pyrroli	DELTA-1-PYRRC	oxidoreductase activity(GO:00			dehydrogenase	5-Hydroxytrypt	1-pyrroline-5-c	proline metabc	mitochond
MOUSE|MGI=I	Q68FE6	Rho family-inte	RHO FAMILY-INTERACTING CELL POLARIZATION REGULATOR 1				non-receptor serine/threonine		14-3-3 protein	Rho protein sig	cytoplasm(
MOUSE|MGI=I	Q9DBL1	Short_branche	SHORT_BRANC	oxidoreductase activity, acting		mitochondrion	dehydrogenase(PC00092)		2-methylbutan	isoleucine cata	mitochond
MOUSE|MGI=I	Q9CQ62	2,4-dienoyl-Co/	2,4-DIENOYL-C(	oxidoreductase	fatty acid beta-	mitochondrion	oxidoreductase(PC00176)		2,4-dienoyl-Co/	lipid metabolic	nucleoplas
MOUSE|MGI=I	P47740	Aldehyde dehy	ALDEHYDE DEH	oxidoreductase	cellular metabc	cytoplasm(GO:	dehydrogenase	5-Hydroxytrypt	3-chloroallyl al	response to rea	cytoplasm(
MOUSE|MGI=I	P51660	Peroxisomal m	PEROXISOMAL	hydro-lyase act	fatty acid beta-oxidation(GO:0006635)				3-hydroxyacyl-(	very long-chair	mitochond
MOUSE|MGI=I	Q61425	Hydroxyacyl-co	HYDROXYACYL-	oxidoreductase	fatty acid beta-oxidation(GO:0006635)				3-hydroxyacyl-(	lipid metabolic	nucleoplas
MOUSE|MGI=I	Q99L13	3-hydroxyisobu	3-HYDROXYISO	oxidoreductase activity, acting on the CH-OH g			dehydrogenase(PC00092)		3-hydroxyisobu	valine catabolic	mitochond
MOUSE|MGI=I	Q8QZS1	3-hydroxyisobu	3-HYDROXYISO	thiolester hydr	alpha-amino acid metabolic pr		hydrolase(PC00121)		3-hydroxyisobu	valine catabolic	mitochond
MOUSE|MGI=I	Q8K354	Carbonyl reduc	CARBONYL RED	oxidoreductase activity, acting on the CH-OH g			reductase(PC00198)		3-keto sterol re	phylloquinone	nucleoplas
MOUSE|MGI=I	O88736	3-keto-steroid r	3-KETO-STEROI	oxidoreductase	sterol metaboli	endoplasmic re	reductase(PC0(	Androgen/estr(	3-keto sterol re	lipid metabolic	endoplasm
MOUSE|MGI=I	Q99LB2	Dehydrogenase	DEHYDROGENA	oxidoreductase activity, acting on the CH-OH g			dehydrogenase(PC00092)		3-keto sterol re	retinal metabo	nucleus(G(
MOUSE|MGI=I	Q9D404	3-oxoacyl-[acyl	3-OXOACYL-[A(	acyltransferase	fatty acid biosynthetic process(GO:0006633)				3-oxoacyl-[acyl	lipid metabolic	mitochond
MOUSE|MGI=I	Q9QXX4	Calcium-bindin	CALCIUM-BIND	L-glutamate tra	C4-dicarboxylate transport(GO		secondary carrier transporter(I		3-sulfino-L-alar	gluconeogenes	mitochond
MOUSE|MGI=I	Q8BH59	Calcium-bindin	CALCIUM-BIND	L-glutamate tra	C4-dicarboxylate transport(GO		secondary carrier transporter(I		3-sulfino-L-alar	glutamate bios	mitochond
MOUSE|MGI=I	Q3TIU4	2',5'-phosphod	2',5'-PHOSPHO	3'-5'-RNA exon	nuclear-transcr	mitochondrion	mRNA polyadenylation factor(I		3'-5'-RNA exon	nuclear-transcr	mitochond

2. To schedule a PRM assay, navigate to Data > Create PRM assay From DIA in the EncyclopeDIA GUI.

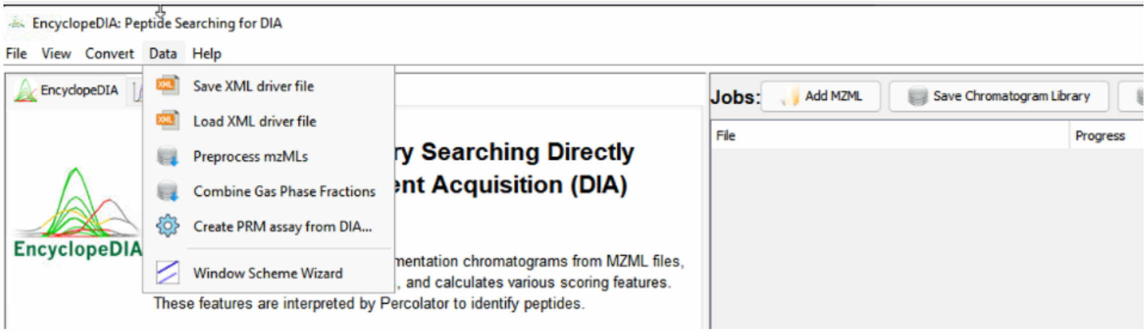

This will pull up the PRM Scheduler window (below).

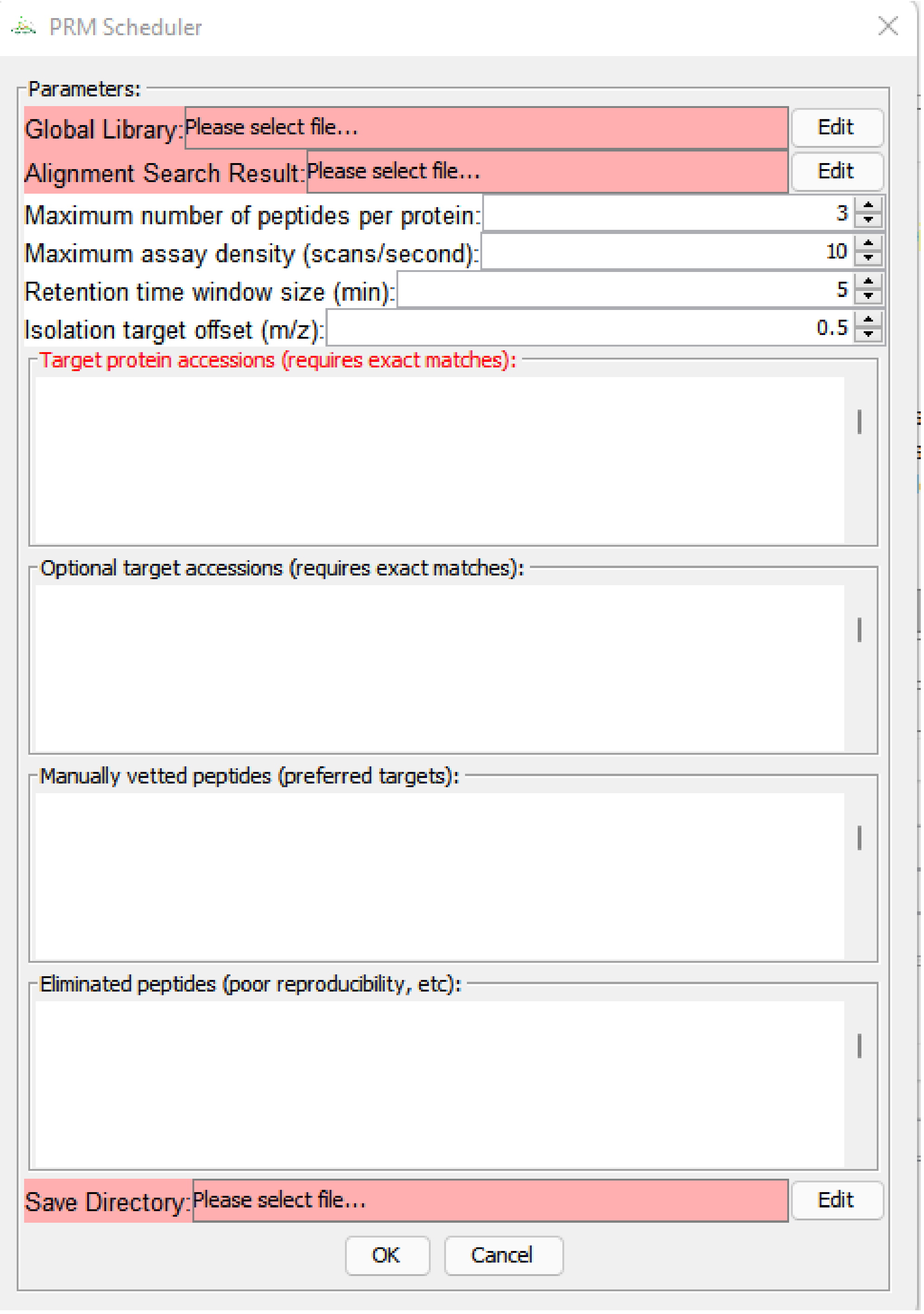

3. Input the translation library under the parameters tab in the slot that says "Global Library." This should be composed of 6 GPF-DIA injections processed in EncyclopeDIA, and acquired on the same instrument that will be used for PRM. The input for this should be a .ELIB. Within this tutorial, we use the "pooled_6xgpf_library," which is composed of pooled IL-2 and IL-15 stimulated T cell proteomes.

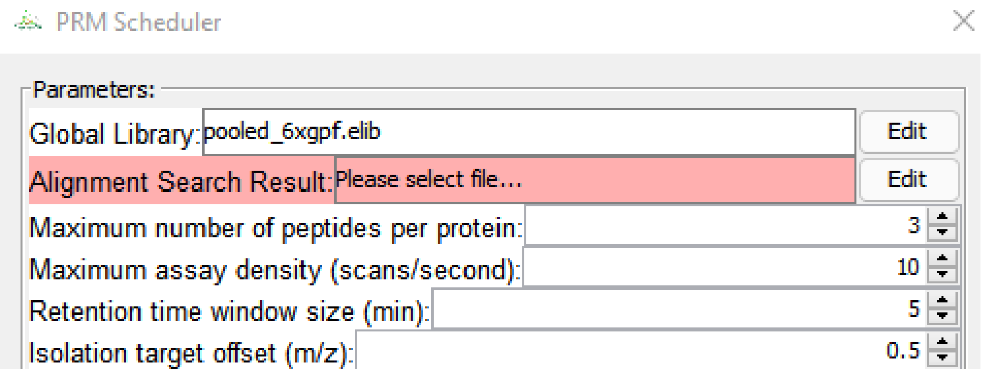

4. Next, input the “Alignment Search Result”, which is a recent DIA (preferably within the past 6-12 hours) injection where retention times will be extracted for target peptides and used for scheduling an assay. The “Alignment Search Result” must be a single DIA injection, processed in EncyclopeDIA using the same chromatography and column setup. In this tutorial, we used an injection containing 8 *m/z* wide isolation windows for our DIA injections. The input should be in the .ELIB format. If the “Alignment Search Result” file is is not from a recent injection, the retention times windows may be off-centered, resulting in truncated peaks.

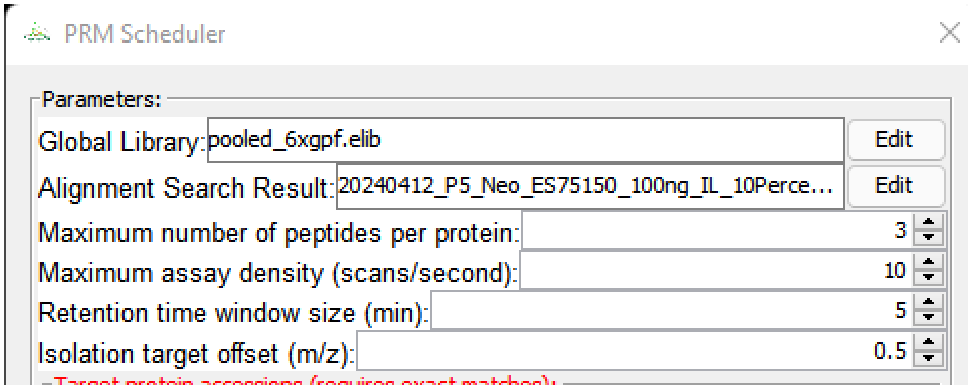

The input shown is a DIA injection acquired with the 10% injection at the 100 ng level, indicating that the sample contained 90% of the background and 10% of the target proteome. The file is named “20240412_P5_Neo_ES75150_100ng_IL_10Percent_DIA_8mz_60min_03.mzML.”

5. Next, set the parameters for the assay. The cycle time determines the number of scans/second, which we refer to as the assay density in the PRM Scheduler GUI. Within this example assay, we are monitoring 50 peptides/cycle to achieve a cycle time of under 2 seconds if the maximum IIT is set to 50 ms.

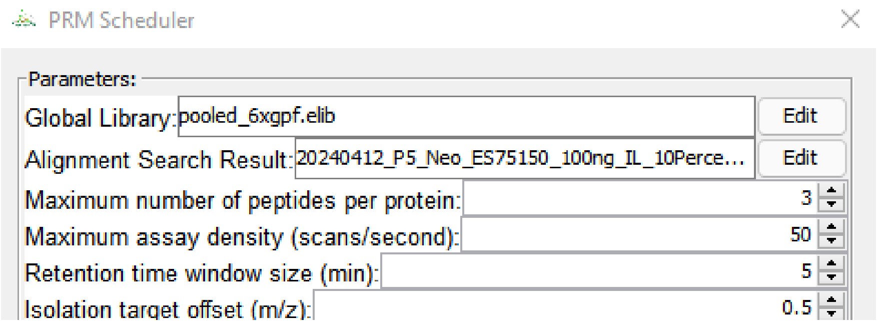

6. The final required input is the target proteins/accession numbers. Paste in the accession numbers you wish to schedule. These are the proteins of interest, and can be determined from Step A. Here, we have input all relevant accession numbers.
   **Note - If inputting accession numbers gives an error in the console, try to put the accession in the format used in the ELIB for proteins, which is sp|ACCESSION|UNIPROT_SPECIES.*

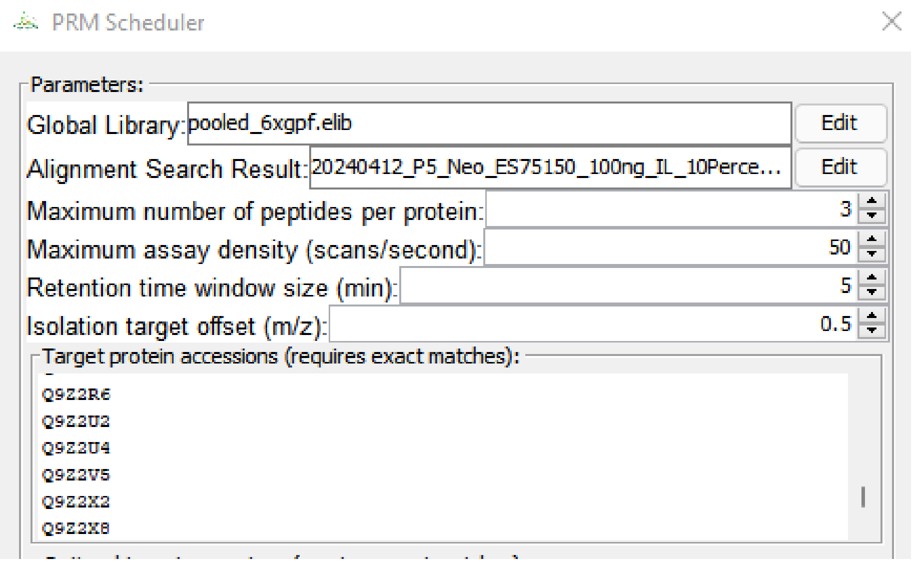

7. The next box can hold "Optional Target Accessions." When scheduling a PRM, the algorithm will attempt to schedule the "Target Protein Accessions" first, then move onto all "Optional Target Accessions" and attempt to schedule each.In this tutorial, we will put the remaining proteins that were detected in our DIA library. The algorithm then selects peptides from our "Optional Target" list of protein accessions, and there is still space in the assay, the optional target accessions will be scheduled.

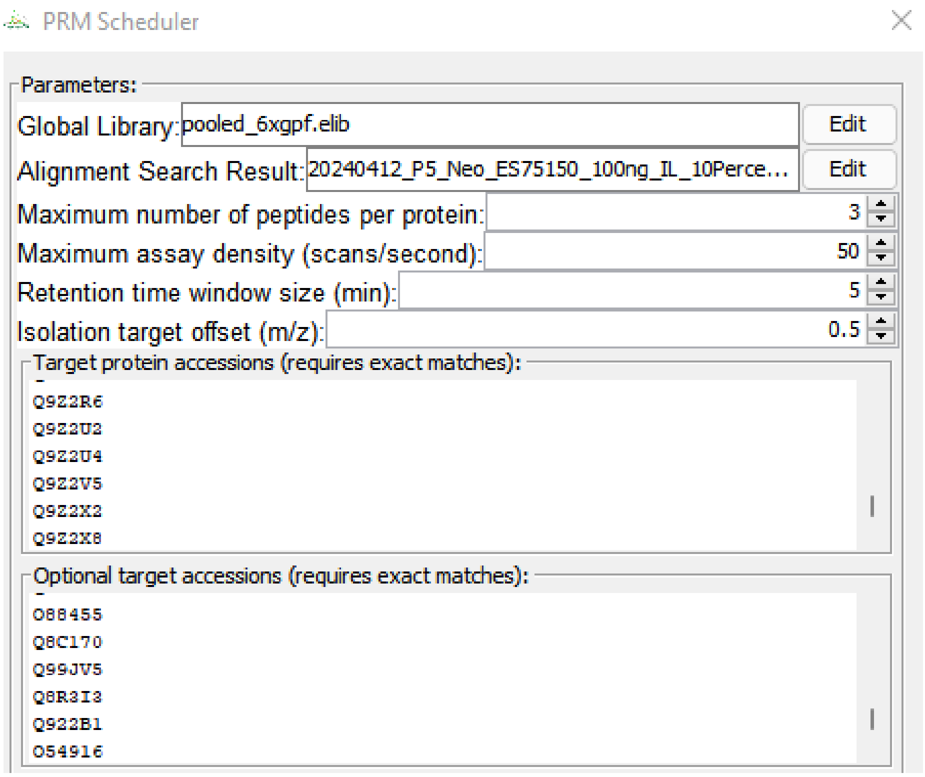

8. Additionally, you can input a list of “manually vetted” peptides. These are peptides that the algorithm will preferentially attempt to schedule first. To determine the peptides to input here, you will want to visualize the chromatograms for proteins you are interested in. For example, you can have manually assessed the peptides that were in the “Target protein accession” and “Optional Protein Accessions,” and select peptides from your translation library that contain low interference or background and clear. For this tutorial, all peptides on the “Target protein accession” and “Optional Protein Accessions” were used as the preferred targets.

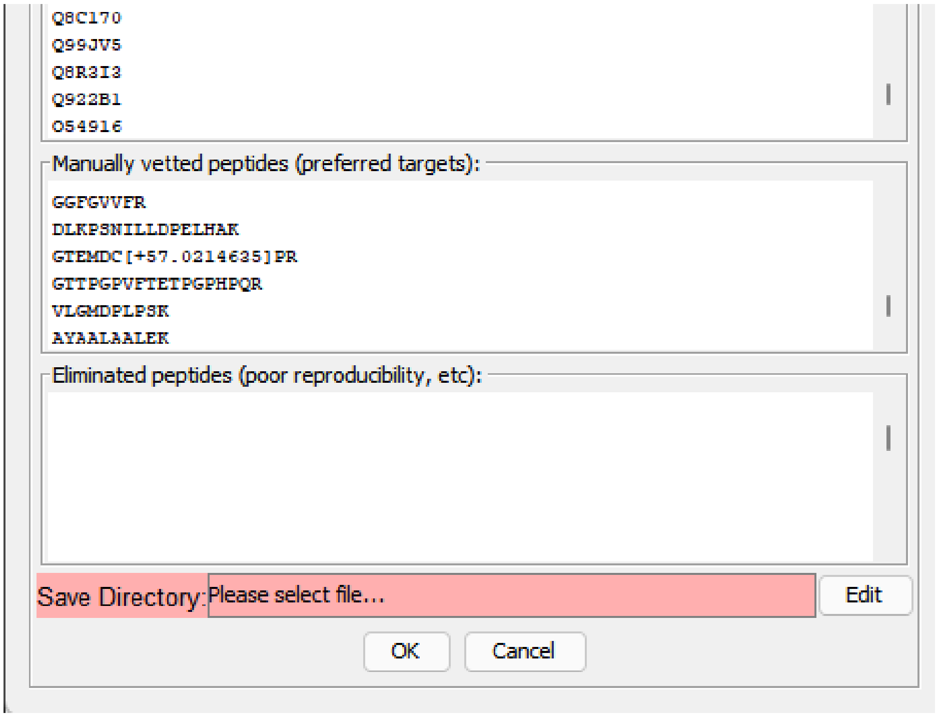

9. Peptides that are not suitable for PRM can be placed either by looking at chromatograms on Skyline or EncyclopeDIA. Place these peptides on an exclusion list.
    a. In this tutorial, we are not using an exclusion list.

10. Finally, name the directory to where the scheduled assay with be output and press “ok” to schedule.

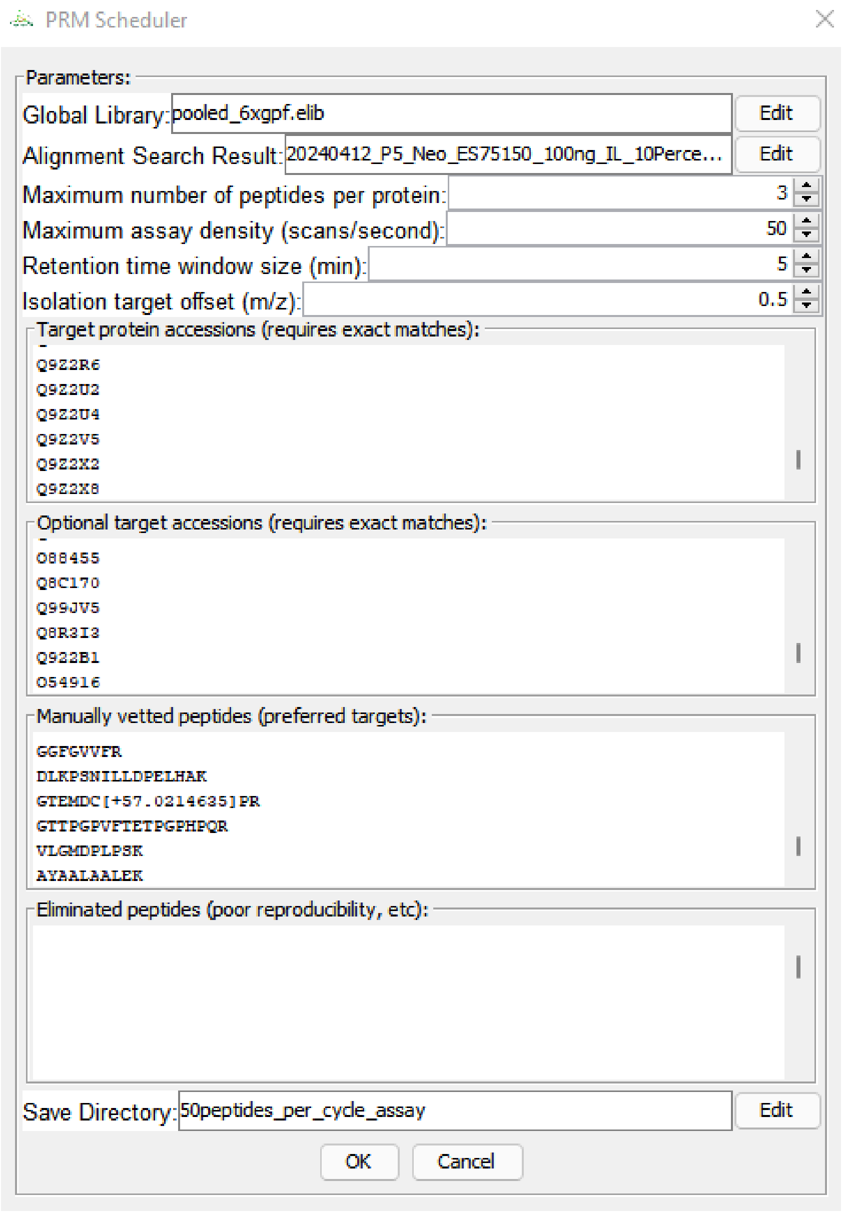

Part B: Outputs from the PRM Scheduler

1. There are several outputs from the PRM Scheduler as follows:

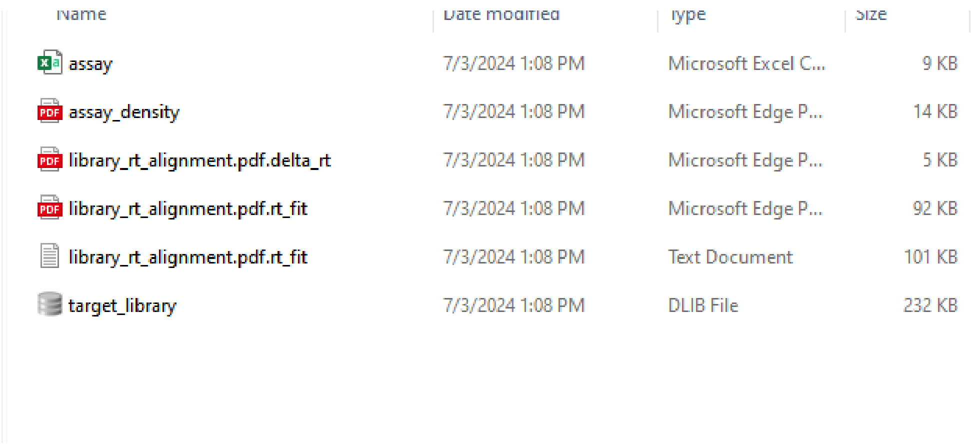

2. The “assay” is output as a .csv file, which is in the format of a Mass List input for scheduling a PRM in Thermo’s Method Editor software. You should be able to upload this file as a mass list to generate a PRM assay easily

.

**Note - If you have trouble uploading the Mass List in Thermo's method editor, check to ensure that the columns are the same in the assay.csv and your method editor. In some versions of the Method Editor, the columns may be arranged or named differently. This software was developed with an experimental version of the Method Editor.*

3. The "assay_density.pdf" shows the number of scheduled peptides over the chromatographic gradient.

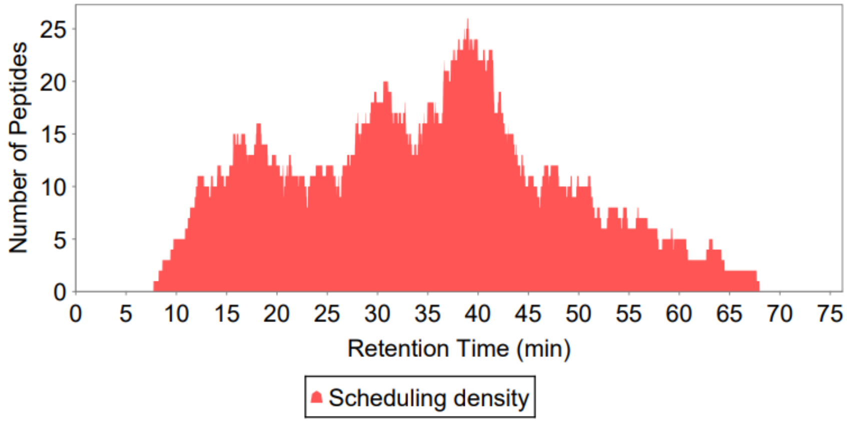

In the case of this assay, a maximum of 25 peptides per cycle were scheduled, however, the schedule still contains space. This is due to the fact that some peptides were not present in the "Alignment Search Result." Peptides must be present "Alignment Search Result" and translation, referred to as the "Global Library" in the GUI, in order to get scheduled.

4. The "library_rt_alignment.pdf.delta.rt_fit.pdf" and the "library_rt_alignment.pdf.rt_fit.pdf" show the alignment between the "Global Library" and the recent injection file ("Alignment Search Result") in an EncyclopeDIA-styled output plot. The x-axis shows the library retention time, while the y-axis shows the actual retention time from the alignment injection. The blue dots indicate peptides that fall in the median retention time, while the red dots are peptides with retention times that differ more than 2 standard deviations away from the median.

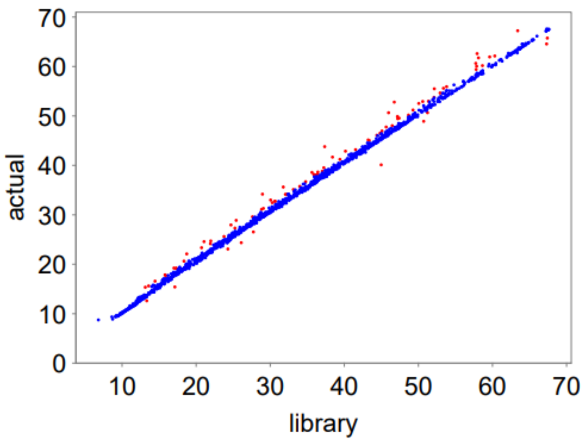

Similarly, the next plot will show changes in retention time and number of peptides containing that retention time shift. If using this as a QC metric, ensure the delta RT from library falls around +/- 2 min. If the Delta RT from library is greater than the "Retention Time Window Size" specified in the assay setup, then retention times have shifted significantly and a new "Alignment Search Result," or recent

DIA injection is needed. Similarly, the “library_rt_alignment.pdf.rt.fit.txt” contains the data that is shown in this plot.

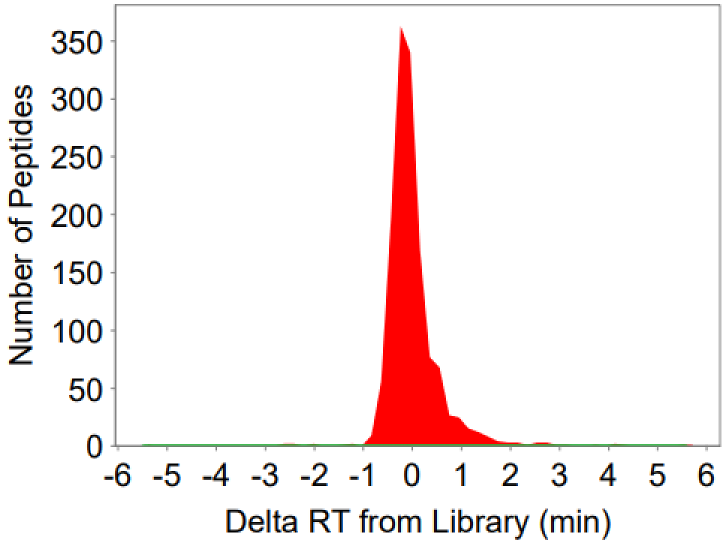

5. The final output is a library named “target_library.DLIB,” which contains all peptides that were scheduled for the assay. This library can be converted to a .BLIB format within EncyclopeDIA by navigating to Convert > Convert Library to BLIB. The .BLIB can be uploaded to Skyline as a library to analyze the PRM data scheduled.

Supplementary References

1. Peters-Clarke, T. M., Coon, J. J. & Riley, N. M. Instrumentation at the leading edge of proteomics. *Anal. Chem.* **96**, 7976–8010 (2024).
2. Heil, L. R., Remes, P. M. & MacCoss, M. J. Comparison of Unit Resolution Versus High-Resolution Accurate Mass for Parallel Reaction Monitoring. *J. Proteome Res.* **20**, 4435–4442 (2021).
3. Phlairaharn, T. *et al.* Optimizing Linear Ion-Trap Data-Independent Acquisition toward Single-Cell Proteomics. *Anal. Chem.* **95**, 9881–9891 (2023).
4. Remes, P. M. *et al.* Hybrid quadrupole mass filter-radial ejection linear ion trap and intelligent data acquisition enable highly multiplex targeted proteomics. *J. Proteome Res.* **23**, 5476–5486 (2024).
