# Supplementary material for: Rapid assay development for low input targeted proteomics using a versatile linear ion trap
Source: Nat Commun. 2025 Apr 23;16:3794. doi: 10.1038/s41467-025-58757-8 (PMC12015518; doi:10.1038/s41467-025-58757-8)
Supplement: Supplementary file 2 — Reporting Summary [file 41467_2025_58757_MOESM2_ESM.pdf]

nature portfolio

Corresponding author(s): Dr. Brian Searle

Last updated by author(s): Mar 11 2025

Reporting Summary

Nature Portfolio wishes to improve the reproducibility of the work that we publish. This form provides structure for consistency and transparency in reporting. For further information on Nature Portfolio policies, see our Editorial Policies and the Editorial Policy Checklist.

Statistics

For all statistical analyses, confirm that the following items are present in the figure legend, table legend, main text, or Methods section.

n/a	Confirmed	
[ ]	[x]	The exact sample size (*n*) for each experimental group/condition, given as a discrete number and unit of measurement
[ ]	[x]	A statement on whether measurements were taken from distinct samples or whether the same sample was measured repeatedly
[x]	[ ]	The statistical test(s) used AND whether they are one- or two-sided<br>*Only common tests should be described solely by name; describe more complex techniques in the Methods section.*
[x]	[ ]	A description of all covariates tested
[ ]	[x]	A description of any assumptions or corrections, such as tests of normality and adjustment for multiple comparisons
[ ]	[x]	A full description of the statistical parameters including central tendency (e.g. means) or other basic estimates (e.g. regression coefficient) AND variation (e.g. standard deviation) or associated estimates of uncertainty (e.g. confidence intervals)
[x]	[ ]	For null hypothesis testing, the test statistic (e.g. *F*, *t*, *r*) with confidence intervals, effect sizes, degrees of freedom and *P* value noted<br>*Give P values as exact values whenever suitable.*
[x]	[ ]	For Bayesian analysis, information on the choice of priors and Markov chain Monte Carlo settings
[x]	[ ]	For hierarchical and complex designs, identification of the appropriate level for tests and full reporting of outcomes
[x]	[ ]	Estimates of effect sizes (e.g. Cohen's *d*, Pearson's *r*), indicating how they were calculated

Our web collection on statistics for biologists contains articles on many of the points above.

Software and code

Policy information about availability of computer code

Data collection	XCalibur v1.0.4283 for mass spectrometry data, SpectraFlo v3 for flow cytometry data
Data analysis	EncyclopeDIA v.4.7.11, Proteome Discoverer 3.1 (CHIMERYS node), Prosit v2019, Skyline 23.1.0.455, FlowJo v10, Proteowizard 3.0.23146

For manuscripts utilizing custom algorithms or software that are central to the research but not yet described in published literature, software must be made available to editors and reviewers. We strongly encourage code deposition in a community repository (e.g. GitHub). See the Nature Portfolio guidelines for submitting code & software for further information.

Data

Policy information about availability of data

All manuscripts must include a data availability statement. This statement should provide the following information, where applicable:

- Accession codes, unique identifiers, or web links for publicly available datasets
- A description of any restrictions on data availability
- For clinical datasets or third party data, please ensure that the statement adheres to our policy

Proteomics data and Skyline documents are available on Panorama at https://panoramaweb.org/StellarIonTrapForLowInput.url. All proteomics raw data is also publicly available on the MASSIVE repository under the accession number MSV000094904 (ftp://massive.ucsd.edu/v08/MSV000094904/). Open-source software developed for this project is publicly available as part of the EncyclopeDIA project at https://bitbucket.org/searleb/encyclopedia.

Research involving human participants, their data, or biological material

Policy information about studies with human participants or human data. See also policy information about sex, gender (identity/presentation), and sexual orientation and race, ethnicity and racism.

Reporting on sex and gender	N/a
Reporting on race, ethnicity, or other socially relevant groupings	N/a
Population characteristics	N/a
Recruitment	N/a
Ethics oversight	The institutional review board at the Ohio State University approved the use of animal models in this study.

Note that full information on the approval of the study protocol must also be provided in the manuscript.

Field-specific reporting

Please select the one below that is the best fit for your research. If you are not sure, read the appropriate sections before making your selection.

☒ Life sciences ☐ Behavioural & social sciences ☐ Ecological, evolutionary & environmental sciences

For a reference copy of the document with all sections, see nature.com/documents/nr-reporting-summary-flat.pdf

Life sciences study design

All studies must disclose on these points even when the disclosure is negative.

Sample size	A sample size of 2 mice were used for the purpose of this study. One biological replicate was used to generate the IL-2 stimulated T cell samples, while the other spleen was used to generate the IL-15 stimulated T cell samples. No stimualted controls were taken from each spleen. For Il-2 and IL-15 stimulated T cells, a total of 90 wells were combined from cell culture into a single replicate for cells stimulated until Day 6, and 46 were pooled for cells stimulated until day 10. 24-well plates were used for cell culture.
Data exclusions	Technical replicates of the PRM data was excluded at the 1 ng level in IL-15 harvested at Day 6 data as a result of an instrument error which caused no sample to be injected. The samples which were blank were excluded from this study.
Replication	Two spleens were used to isolate immune cells for culture. Cells were expanded into 90 wells for an earlier time point, and 46 wells for a later time point, then combined for mass spectrometry. 1 representative well from each condition was used for flow cytometry on days 5, 6 and 10. PRM assays at the 1 ng level on IL-15 and IL-2 stimulated T cell proteomes, harvested at Days 6 and 10, were collected in triplicate. The analogous 10 ng and 100 ng assays were collected in duplicate. All libraries and calibration curves were not replicated.
Randomization	For PRM technical replicates, the IL-2 and IL-15 stimulated T cell proteomes harvested at Days 6 and 10 were randomized in their injection order. One technical replicate of each condition was blocked together and acquired prior to acquiring second and third technical replicates.
Blinding	Not applicable.

Reporting for specific materials, systems and methods

We require information from authors about some types of materials, experimental systems and methods used in many studies. Here, indicate whether each material, system or method listed is relevant to your study. If you are not sure if a list item applies to your research, read the appropriate section before selecting a response.

Materials & experimental systems

n/a	Involved in the study	
☐	☒	Antibodies
☒	☐	Eukaryotic cell lines
☒	☐	Palaeontology and archaeology
☐	☒	Animals and other organisms
☒	☐	Clinical data
☒	☐	Dual use research of concern
☒	☐	Plants

Methods

n/a	Involved in the study	
☒	☐	ChIP-seq
☐	☒	Flow cytometry
☒	☐	MRI-based neuroimaging

Antibodies

Antibodies used	Antibody information is listed in supplemental data Table S1 in the supplemental PDF.
Validation	The antibodies for B220 and CD25 from ThermoFisher Scientific underwent advanced verification, according to the manufacturer's website. All other antibodies were verified using isoype controls by the manufacturers.

Animals and other research organisms

Policy information about studies involving animals; ARRIVE guidelines recommended for reporting animal research, and Sex and Gender in Research

Laboratory animals	Female mice from the C57BL/6J strain were sacrificed for this work at the age of 4 months and 11 days.
Wild animals	N/a
Reporting on sex	Sex was not considered in regards to this study. All biology that is discussed is reported in prior literature, therefore we do not consider that sex is a covariate in this study.
Field-collected samples	N/a
Ethics oversight	The use of an animal murine model was approved by the Institutional Review Board at the Ohio State University.

Note that full information on the approval of the study protocol must also be provided in the manuscript.

Plants

Seed stocks	N/a
Novel plant genotypes	N/a
Authentication	N/a

Flow Cytometry

Plots

Confirm that:

- [x] The axis labels state the marker and fluorochrome used (e.g. CD4-FITC).
- [x] The axis scales are clearly visible. Include numbers along axes only for bottom left plot of group (a 'group' is an analysis of identical markers).
- [x] All plots are contour plots with outliers or pseudocolor plots.
- [x] A numerical value for number of cells or percentage (with statistics) is provided.

Methodology

Sample preparation	On days 5, 6, and 10, flow cytometry was performed with the following procedure: cultures pooled together, washed three times with PBS, and counted on a hemocytometer. Cells were then stained in the dark for 30 minutes at 4℃ with the viability dye (Live/Dead Fixable Blue Dead Cell Stain kit, for UV excitation from Thermo Fisher Scientific) at a ratio of 1:1000. Extracellular markers, anti-CD4, anti-CD45R, anti-CD8, and anti-TCR-β at a concentration of 1 to 400 antibody to cell solution. Additionally, anti-CD69, anti-CD62L, and anti-CD25 were added at a 1 to 200 ratio. Anti-CD44 was applied at a ratio of 1 to 600, and the cell solution was 20 minutes at 4℃. More details on the antibody panel are available in Supplemental Table 1. Stained cells were analyzed with a Cytek Biosciences Aurora 5-laser flow cytometer. Data was processed and visualized in BD Biosciences FlowJo™ software.
Instrument	Cytek Biosciences Aurora System
Software	BD Biosciences FlowJo Software was used for analysis. The SpectraFlo software from Cytek Biosciences was used to acquire data on the flow cytometer.

Cell population abundance	On day 6, there were 92.5 million IL-15 stimulated T cells and 119.5 million T cells for the IL-2 condition. On day 10, there were 153 million IL-15 cells stimulated T cells, and 90.5 million Il-2 stimulated T cells. Cells were counted using a hemocytometer, and purity was determined using flow cytometry. On day 6, 39.2% of the IL-15 culture were lymphocytes, while 48% of the culture were lymphocytes in the IL-2 stimulated condition. On day 10 68.5% of the IL-15 culture were lymphocytes, while 54.1% of the culture were lymphocytes. Flow cytometry without cell sorting was performed, therefore the purity of each population was not checked.
Gating strategy	In each culture and time point, lymphocytes were gaited on FSC-A (forwrad scattering area) and SSC-A (side-scattering area), then single cells were selected using FSC-W and FSC-A, then by SSC-W (side-scatering width) and FSC-A. Viability was determined using a Live/Dead Blue A Viability stain against FSC-A. T cells were gaited using TCR-Beta and B220.

☒ Tick this box to confirm that a figure exemplifying the gating strategy is provided in the Supplementary Information.
